# Regulatory effects of silicon nanoparticles on the growth and photosynthesis of cotton seedlings under salt and low-temperature dual stress

**DOI:** 10.1186/s12870-023-04509-z

**Published:** 2023-10-21

**Authors:** Yueping Liang, Hao Liu, Yuanyuan Fu, Penghui Li, Shuang Li, Yang Gao

**Affiliations:** 1 Institute of Farmland Irrigation, Chinese Academy of Agricultural Sciences, Xinxiang, 453002 China; 2 College of Agronomy, Tarim University, Alaer, 843300 China; 3 Shandong Academy of Agricultural Machinery Science, Shandong Academy of Agricultural Sciences, Jinan, 250100 China

**Keywords:** Cotton seedlings, Photosynthesis, Regulatory mechanism, Salt and low-temperature dual stress, Silicon nanoparticles

## Abstract

**Background:**

Silicon nanoparticles (SiO_2_-NPs) play a crucial role in plants mitigating abiotic stress. However, the regulatory mechanism of SiO_2_-NPs in response to multiple stress remains unclear. The objectives of this study were to reveal the regulatory mechanism of SiO_2_-NPs on the growth and photosynthesis in cotton seedlings under salt and low-temperature dual stress. It will provide a theoretical basis for perfecting the mechanism of crop resistance and developing the technology of cotton seedling preservation and stable yield in arid and high salt areas.

**Results:**

The results showed that the salt and low-temperature dual stress markedly decreased the plant height, leaf area, and aboveground biomass of cotton seedlings by 9.58%, 15.76%, and 39.80%, respectively. While SiO_2_-NPs alleviated the damage of the dual stress to cotton seedling growth. In addition to reduced intercellular CO_2_ concentration, SiO_2_-NPs significantly improved the photosynthetic rate, stomatal conductance, and transpiration rate of cotton seedling leaves. Additionally, stomatal length, stomatal width, and stomatal density increased with the increase in SiO_2_-NPs concentration. Notably, SiO_2_-NPs not only enhanced chlorophyll a, chlorophyll b, and total chlorophyll content, but also slowed the decrease of maximum photochemical efficiency, actual photochemical efficiency, photochemical quenching of variable chlorophyll, and the increase in non-photochemical quenching. Moreover, SiO_2_-NPs enhanced the activities of ribulose-1,5-bisphosphate carboxylase/oxygenase and phosphoenolpyruvate carboxylase, improved leaf water potential, and decreased abscisic acid and malondialdehyde content. All the parameters obtained the optimal effects at a SiO_2_-NPs concentration of 100 mg L^− 1^, and significantly increased the plant height, leaf area, and aboveground biomass by 7.68%, 5.37%, and 43.00%, respectively. Furthermore, significant correlation relationships were observed between photosynthetic rate and stomatal conductance, stomatal length, stomatal width, stomatal density, chlorophyll content, maximum photochemical efficiency, actual photochemical efficiency, photochemical quenching of variable chlorophyll, and Rubisco activity.

**Conclusion:**

The results suggested that the SiO_2_-NPs improved the growth and photosynthesis of cotton seedlings might mainly result from regulating the stomatal state, improving the light energy utilization efficiency and electron transport activity of PSII reaction center, and inducing the increase of Rubisco activity to enhance carbon assimilation under the salt and low-temperature dual stress.

## Introduction

Soil salinization has become an important global issue regarding resources and ecological environments [1]. Additionally, it is one of the main factors limiting crop growth, development, and the increase in stable yield, which poses a severe threat to global food security [2, 3]. Soil salinization is getting worse worldwide, and about 50% of the total agricultural land is expected to be covered by saline soils by 2050 due to climate change and irrational water use for agriculture [4]. Meanwhile, with the gradual increase of the global greenhouse effect, extreme temperature events such as cold waves occur frequently [5], and the probability of crops suffering from low-temperature stress becomes more and more severe in the 21st century [6]. Salt and low-temperature stress lead to shared or specific damage to plants, including osmotic stress, oxidative stress, nutritional disorders, reduced activities of various functional enzymes, alteration of photosynthetic and metabolic processes, and genotoxicity [7, 8]. Differently, salt stress results in a toxic concentration of Na^+^ and Cl^−^ [9]. Moreover, over accumulation of reactive oxygen species (ROS) negatively affects cell membrane integrity and permeability, ion transport, hormone balance, respiration, oxidation/reduction, DNA and protein synthesis [10, 11]. Together, these subsequently affect the crops growth, development, and yield.

Plants have developed a range of resistant mechanisms to counter salt and low temperature stress. For example, many kinds of osmolytes such as betaines, sorbitol, trehalose, and proline are produced to sustain cellular structures and diminish ROS-induced oxidative injuries [12]. Antioxidant systems such as superoxide dismutase enzymes (SOD), peroxidase enzyme (POD), and catalase (CAT) are activated to scavenge ROS [13]. Abscisic acid (ABA) content is upregulated, affecting the stomatal closure and reducing water loss [14]. Moreover, a transient increase in cytosolic calcium (Ca^2+^) level induces a change in aquaporin abundance/activity allowing the maintenance of the water balance and photosynthesis adjustment [15, 16].

Cotton is an important strategic reserve material in China, and Xinjiang is the main producing area [17, 18], but the growth and yield formation of cotton in this region is seriously threatened by soil salinization [19]. Meanwhile, the area frequently suffers from unpredictable climates such as cold and low-temperature stress during the cotton seedling stage, especially in the southern region of Xinjiang. The metabolic and physiological processes of cotton seedlings are strongly affected by the combination of salt stress and low temperature, which leads to serious damage to cotton growth, yield, and quality.

Photosynthesis provides materials and energy for plant growth, development, and yield formation [20, 21], and it is one of the physiological processes that are significantly affected by salt and low-temperature stress [22, 23]. Osmotic stress and excessive accumulation of ROS caused by salt and low temperature stress inhibited photosynthesis via stomatal factors and non-stomatal factors [24]. The former is mainly the result of reducing stomatal aperture or stomatal density or irregular distribution of stomatal spaces, which leads to a decrease in CO_2_ concentration entering the leaves [25]. Among non-stomatal factors, salt and low temperature stresses accelerate the degradation of photosynthetic pigment and the destruction of the chloroplast structure and interfere with CO_2_ fixation enzymes such as phosphoenolpyruvate carboxylase (PEPC) and ribulose diphosphate carboxylase (Rubisco) [26], which result in an excessive production of ROS in photosynthetic tissues. Moreover, an increase in the non-photochemical quenching (*NPQ*) to reduce damage to the photosynthetic apparatus, and a decrease in the actual photochemical efficiency of photosystem II (*Φ*_*PSII*_), maximal photochemical efficiency of photosystem (*Fv/Fm*), and photochemical quenching of variable chlorophyll (*qP*) indicate the light energy utilization is restricted and electron transport chain of photosynthesis is damaged [27]. Eventually, it leads to the inhibition of plant growth and development. Therefore, it is imperative to develop technical measures to alleviate stress damage and enhance the photosynthesis of cotton seedlings under salt and low temperature dual stress, and it is of great significance for ensuring healthy and robust growth of cotton seedlings and achieving stable cotton yield.

Silicon is a beneficial element for plant growth and development and has a significant role in mitigating abiotic stresses such as salt, drought, and low-temperature stress [28, 29]. With the rapid development of nanotechnology, the scope of its application in plants and agriculture is expanding. Nanoscale silicon particles (SiO_2_-NPs) not only possess superior magnetoelectric, mechanical, and thermodynamic properties compared to ordinary silicon, but also have a smaller particle size, larger surface area, energy, and binding capacity [30, 31], which results in their ease of absorption and participation in the physiological metabolism of plants to alleviate the harm of abiotic stress on plant growth [32–34]. Previous studies have demonstrated that exogenous application of SiO_2_-NPs could alleviate the damage of abiotic stress by increasing osmotic regulatory substances, reducing electrolyte leakage and malondialdehyde content, scavenging the toxic effects of ROS, decreasing excessive uptake of Na^+^ and other approaches, which caused an increase in leaf water content, stomatal conductance, chlorophyll content, PSII activity and photosynthetic enzyme activity. Ultimately, it resulted in the improvement of the photosynthesis and growth of crops [35–37]. However, the regulatory effects of SiO_2_-NPs on photosynthesis processes is focused on a single salt or low-temperature stress in previous research [33, 36], the regulatory effects on photosynthesis processes under salt and low-temperature dual stress have not been sufficiently elucidated and need further evaluation.

Therefore, this study focused on cotton seedlings and investigated the responses of the growth, leaf water potential, gas exchange parameters, stomatal characteristics, chlorophyll content, chlorophyll fluorescence characteristics, photosynthetic enzyme activities, abscisic acid and malonaldehyde content to SiO_2_-NPs under salt and low-temperature dual stress. We hypothesized that: (I) Salt and low-temperature dual stress limited the growth and photosynthesis of cotton seedlings. (II) SiO_2_-NPs improved the photosynthesis and growth through the changing of stomatal state, light energy utilization efficiency and electron transport activity of PSII, and photosynthetic enzymes activity under salt and low-temperature dual stress. The objectives of this study were to reveal the regulatory mechanism of SiO_2_-NPs on the growth and photosynthesis in cotton seedlings under salt and low-temperature dual stress. It will provide a theoretical basis for perfecting the mechanism of crop resistance and exploring new technology for cotton seedling preservation and stable yield in arid and high salt areas.

## Materials and methods

### Experimental design

The experiment was conducted in a phytotron in the Xinxiang Experimental Station of the Chinese Academy of Agricultural Sciences (35.09°N, 113.48°E). The phytotron was controlled with a relative humidity of 50–60% and a light period of 12 h (08:00–20:00). The light intensity was maintained at 600 µmol m^− 2^ s^− 1^. Salt and low-temperature dual stress was applied with a salt concentration of 150 mM and temperature of 15 ℃/10 ℃ (day/night). Four concentrations of SiO_2_-NPs (Beike Nano Technology Co., LTD, Suzhou, China) were set at 0 mg L^− 1^ (distilled water), 50 mg L^− 1^, 100 mg L^− 1^ and 200 mg L^− 1^, signed as T1, T2, T3, and T4, respectively. No salt, normal temperature and sprayed distilled water were used as control (CK).

Full and uniformly sized seeds of *Gossypium hirsutum L.*, cultivar Xinluzhong-37 (Talimu River Seed Industry Co., LTD, Xinjiang, China), were disinfected and sown in PVC pots (6 cm diameter, 24 cm height) with drainage holes at the bottom. Each pot was filled with 290 g substrate (Pindstrup Mosebrug A/S, Denmark), three seeds were sown in each pot for germination and growth in the phytotron under 25 °C/20°C (day/night) temperature, and each treatment replicated 12 times. Once the cotton seedlings had fully expanded to two cotyledons, they were thinned to one plant per pot. When the seedlings grew to the first leaf stage, they were irrigated with 80 mL of Hoagland solution every 7 days. Further, when the seedlings grew to the second leaf with one bud stage, they were transferred to the low-temperature phytotron and irrigated with the salt solution on the 1^st^, 4^th^, and 9^th^ day. Simultaneously, different concentrations of SiO_2_-NPs were sprayed on the same day as irrigating with the salt solution on the cotton leaves at a rate of 10 mL per plant. On the 10^th^ day, the latest fully expanded leaves were sampled for measurements.

### Measurements

#### Plant height, leaf area, and aboveground biomass

Plant height was defined by the distance from the soil surface to the highest point of the cotton plant. Leaf area was measured using a portable LI-3000 C leaf area meter (Li-Cor Inc., Lincoln, NE, USA). Three cotton plants were sampled and the roots’ integrity was ensured as much as possible. The plants were thoroughly rinsed and then placed in an oven initially set to 105 ℃ for 30 min to terminate the metabolic activity. Subsequently, the oven was adjusted to 75 ℃, and the plants were dried for 24 h. Finally, the dry weight of the plants was determined using a precision balance accurate to one part in ten thousand.

#### Leaf water potential

The leaf samples were taken between 08:00–09:00 a.m. and stored in self-sealing bags and placed in foam-insulated boxes with ice packs for measurement. Leaf water potential (LWP) was determined using a WP4C (Decagon, Pullman, WA, USA), dewpoint water potential meter [23].

#### Gas exchange parameters

Gas exchange parameters were measured using the Li-COR 6400XT photosynthesis measurement system (Li-COR Inc., Lincoln, NE, USA) with a red and blue light source in the leaf chamber. The photosynthetically active radiation (PAR) was adjusted at 1000 µmol m^− 2^ s^− 1^, and the reference CO_2_ concentration was set at 400 µmol mol^− 1^. Photosynthetic rate (*Pn*), stomatal conductance (*gs*), intercellular CO_2_ concentration (*Ci*), and transpiration rate (*Tr*) of cotton seedlings with fully expanded leaves were measured between 09:00–11:00 a.m.

#### Chlorophyll content

The contents of chlorophyll a, chlorophyll b, and total chlorophyll were measured using the spectrophotometrically method [38]. For the sample preparation a leaf disc was cut, and the chlorophyll was extracted with 80% acetone, the filtrate was measured by spectrophotometer at 663 nm and 645 nm. Chlorophyll content was calculated using the following formula:

1 $$Chlorophyll{\text{ }}a\,\left( {Chl{\text{ }}a} \right) = \left( {12.7 \times D663{\text{ }}-{\text{ }}2.69 \times D645} \right)$$

2 $$Chlorophyll{\text{ }}b{\text{ }}\left( {Chl{\text{ }}b} \right){\text{ }} = {\text{ }}\left( {22.9{\text{ }} \times {\text{ }}D645{\text{ }}-{\text{ }}4.64{\text{ }} \times {\text{ }}D663} \right)$$

3 $$Total\,Chlorophyll{\text{ }}\left( {Chl{\text{ }}t} \right){\text{ }} = {\text{ }}20.2{\text{ }} \times {\text{ }}D645{\text{ }} + {\text{ }}8.02{\text{ }} \times {\text{ }}D663$$

#### Chlorophyll fluorescence parameters

Chlorophyll fluorescence parameters were simultaneously measured on the same day as measuring leaf gas exchange parameters using the ultra-portable modulated chlorophyll fluorometer (MINI-PAM-II, WALZ, Germany). The leaves were adapted to darkness overnight to measure the initial (*Fo*), maximum (*Fm*), variable (*Fv*), and steady-state fluorescence (*Fs*). Additionally, the parameters of maximum (*F’m*) and minimum fluorescence (*F’o*) under light adaptation were determined. The fluorescence parameters were calculated as follows [39]:

4 $$ Fv/Fm = \left( {Fm - Fo} \right)/Fm  $$

5 $${\Phi _{PSII}} = \left( {F'm - Fs} \right)/F'm$$

6 $$qP = \left( {F'm - Fs} \right)/\left( {F'm - F'o} \right)$$

7 $$NPQ = \left( {Fm - F'm} \right)/F'm$$

where *Fv/Fm* is the maximum photochemical efficiency, *Φ*_*PSII*_ is the actual photochemical efficiency, *qP* is the photochemical quenching of variable chlorophyll, and *NPQ* is the non-photochemical quenching.

#### Stomatal characteristics

Stomatal samples were collected using the imprint method and prepared as temporary slides. The stomatal structure was observed and photographed under the Teelen xsp 360a (Teelen Inc., Shanghai, China) microscope with a 40 × objective. The stomatal density (SD) was the number of stomata distributed per unit leaf area, which was recorded under the 10x objective [40]. The stomatal length (SL) and width (SW) were measured separately using ImageJ software (National Institutes of Health, Bethesda, MD, USA).

#### Photosynthesis enzymes and abscisic acid

Cotton leaf samples were frozen immediately in liquid nitrogen for 30 s after collection. The frozen samples were stored in a refrigerator at -80 ℃ for the determination of abscisic acid (ABA) content and the activities of photosynthetic enzymes including phosphoenolpyruvate carboxylase (PEPC) and ribulose diphosphate carboxylase (Rubisco).

ABA was extracted using an isopropanol/water/hydrochloric acid solution [41]. Acid was added to the extract to improve the solubility of the hormone in an organic solvent and to inactivate some enzymes in the tissue. The samples were concentrated using a dichloromethane extraction followed by nitrogen gas blowing. A high-performance liquid chromatography system (ACHROM S3000, Acchrom Tech, Beijing, China) was used to measure the ABA content. Compass-C18 (4.6 mm × 250 mm, 5 μm) chromatographic column was used. The mobile phase was prepared by mixing 600 mL of ultrapure water with 6 mL of acetic acid and then adding 400 mL of methanol. The injection volume was 10 µL, the flow rate was 0.8 mL min^− 1^, the column temperature was 35 ℃ and the total run time was 40 min. The UV detection wavelength was set at 254 nm.

PEPC and Rubisco were extracted by 0.1 g leave tissue and coarsely ground in a mortar under liquid nitrogen with adding 1mL extraction buffer. Soluble and insoluble fractions were separated by centrifugation at 8000 r at 4 ℃ for 10 min, and the supernatant was used to determine enzyme activities. PEPC and Rubisco activities were determined using the colorimetric method with UV detection set at 340 nm [42]. Suzhou Comin Biotechnology Co., Ltd provided the kits used for measurement.

#### Malondialdehyde

Approximately 0.5 g of fresh leaf was ground into a homogenate with 5 mL of 10% trichloroacetic acid solution. The mixture was centrifuged at 8000 rpm for 10 min. 2 mL of the supernatant mixed with 2.0 mL of 0.5% thiobarbituric acid solution were reacted in boiling water for 15 min. After the mixture cooled to room temperature. The absorption of the mixture at 450 nm, 532 nm, and 600 nm was measured by a UV-VIS spectrophotometer. Malondialdehyde (MDA) content was then calculated using the following formula [43]:

8 $$MDA{\text{ }}content{\text{ }} = {\text{ }}6.45{\text{ }} \times {\text{ }}\left( {D532 - D600} \right) - 0.56 \times D450$$

### Statistical analysis

Microsoft Excel 2019 (Microsoft, Redmond, WA, USA) and Origin 8.5 software (Origin Lab, Northampton, MA, USA) were used for data processing and graph plotting, respectively. A one-way analysis of variance (ANOVA) was conducted with all data performed as mean (*n* = 3) followed by standard deviation. Further the least significant difference (LSD) test was adopted for multiple comparisons (*p <* 0.05 and *p <* 0.01) using SPSS Statistics 22 (IBM SPSS Statistics, Chicago, IL, USA) software. Pearson’s correlation coefficient (r) was used to test the correlation between variables and principal component analysis (PCA) was used to assess the relationships among all parameters in CANOCO 5 (Microcomputer Power, Ithaca, NY, USA).

## Results

### Effect of SiO_2_-NPs on plant height, leaf area, and aboveground biomass of cotton seedlings under the salt and low-temperature dual stress

**Fig. 1:**
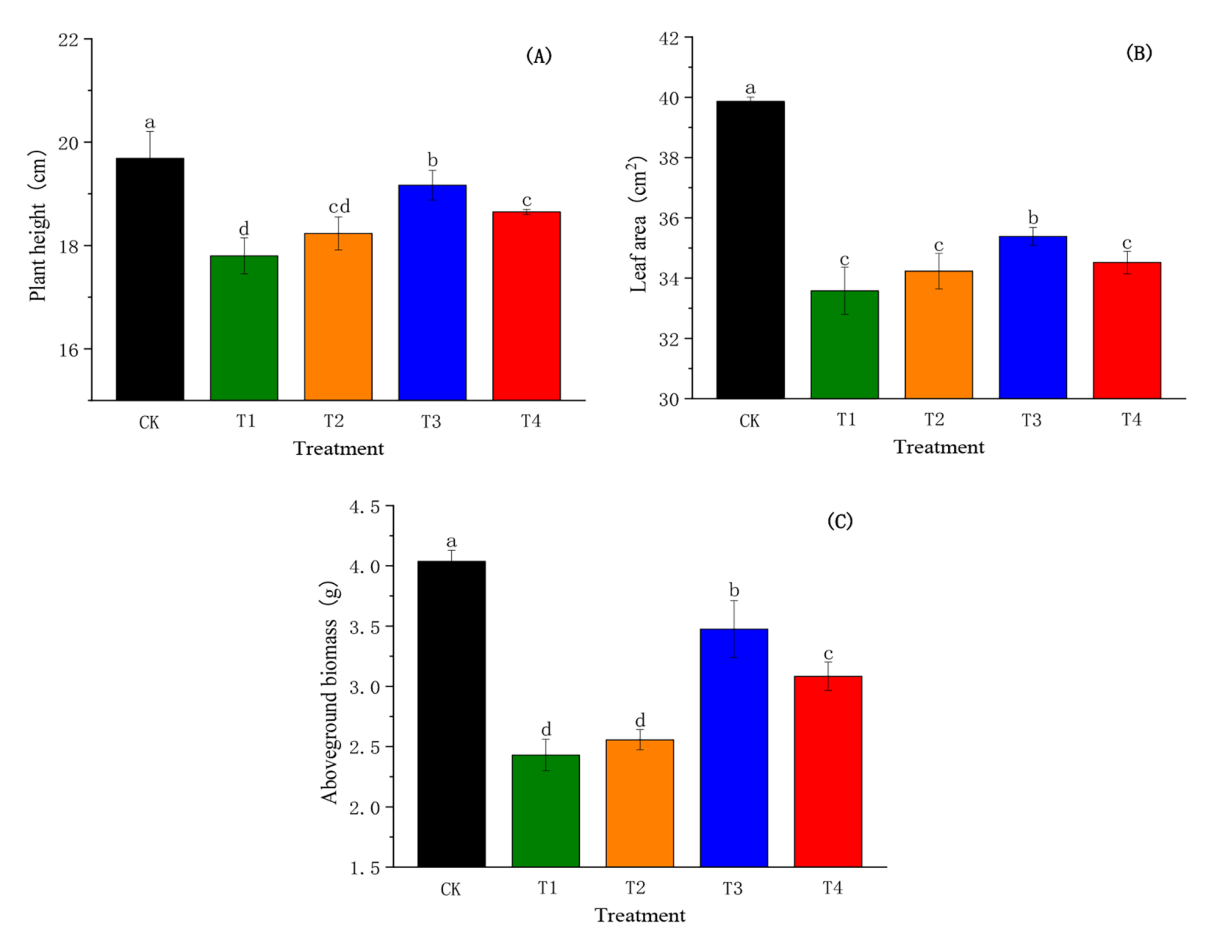
Effects of SiO_2_-NPs on plant height **(A)**, leaf area **(B)**, and aboveground biomass **(C)** of cotton seedlings under the salt and low temperature dual stress. CK represents the foliar application of 0 mg L^− 1^ SiO_2_-NPs with no stress. T1, T2, T3, and T4 represent the foliar application of 0 mg L^− 1^, 50 mg L^− 1^, 100 mg L^− 1^, and 200 mg L^− 1^ SiO_2_-NPs under the salt and low-temperature dual stress, respectively. Data are mean ± standard deviation (*n *= 3). Different alphabets on top of error bars represent significant differences (*p <* 0.05)

The salt and low-temperature dual stress caused an obvious decrease in the plant height, leaf area, and aboveground biomass of cotton seedlings as presented in Fig. 1. The plant height, leaf area, and aboveground biomass in T1 significantly reduced (*p* < 0.05) by 9.58%, 15.76%, and 39.80% compared to CK, respectively. Conversely, SiO_2_-NPs markedly enhanced the plant height, leaf area, and aboveground biomass, and obtained the optimal effects at T3. However, there was no significant difference between T2 and T1.

### Effects of SiO_2_-NPs on gas exchange parameters of cotton seedlings under the salt and low-temperature dual stress

**Fig. 2:**
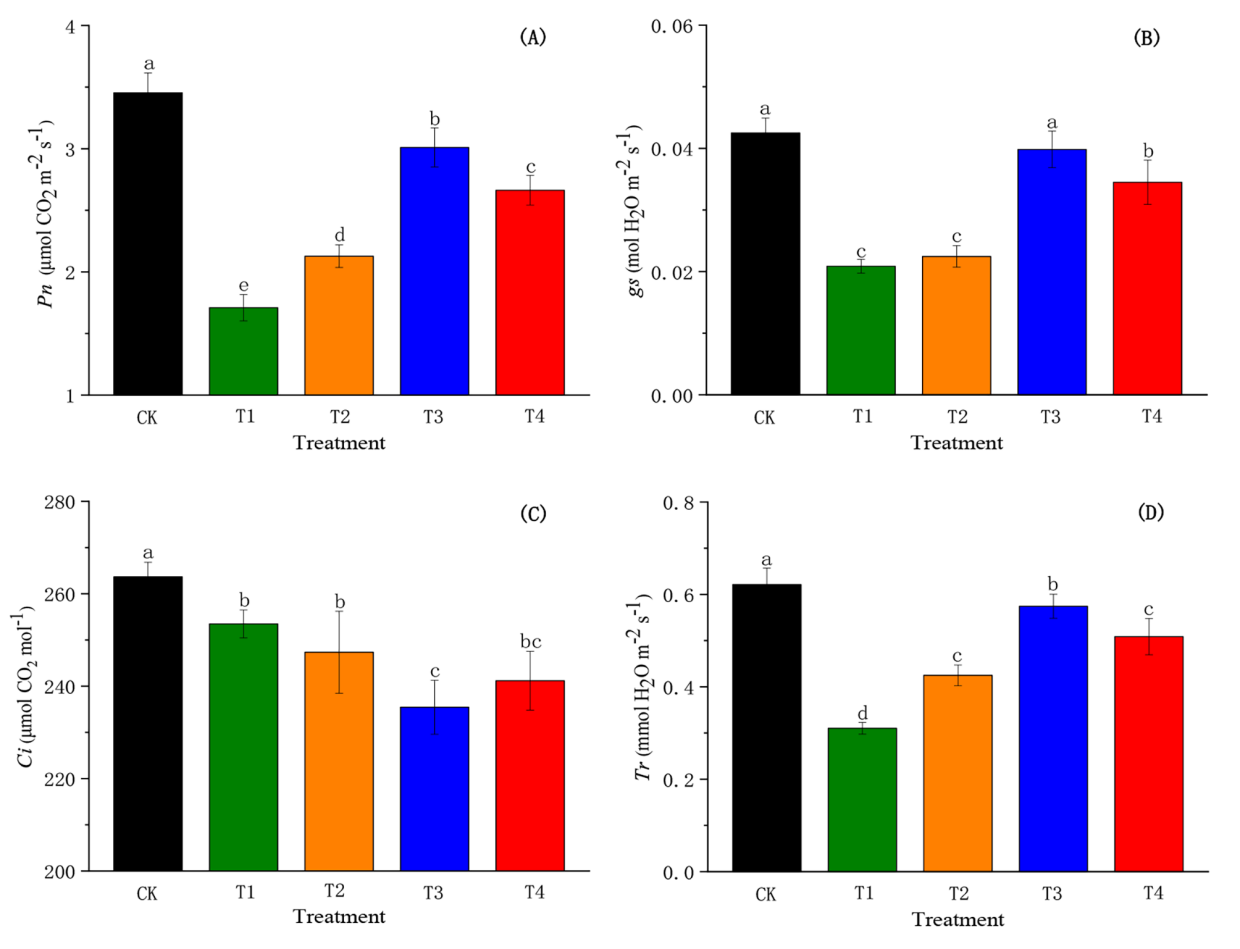
Effects of SiO_2_-NPs on photosynthetic rate (*Pn*, **A)**, stomatal conductance (*gs*, **B)**, intracellular CO_2_ concentration (*Ci*, **C)**, and transpiration rate (*Tr*, **D)** of cotton seedlings under the salt and low temperature dual stress. CK represents the foliar application of 0 mg L^− 1^ SiO_2_-NPs with no stress. T1, T2, T3, and T4 represent the foliar application of 0 mg L^− 1^, 50 mg L^− 1^, 100 mg L^− 1^, and 200 mg L^− 1^ SiO_2_-NPs under the salt and low-temperature dual stress, respectively. Data are mean ± standard deviation (*n* = 3). Different alphabets on top of error bars represent significant differences (*p <* 0.05)

The salt and low-temperature dual stress significantly decreased the *Pn*, *gs*, *Ci*, and *Tr* of cotton seedlings compared to CK (Fig. 2). However, SiO_2_-NPs significantly improved (*p* < 0.05) the *Pn*, *gs*, and *Tr* (Fig. 2A, B, and D) and displayed a similar trend that was first increasing and then decreasing. The *Pn*, *gs*, and *Tr* in T2, T3, and T4 significantly increased (*p <* 0.05) compared to T1, except for the *gs*, whereby no significant differences were observed between T2 and T1. In addition, SiO_2_-NPs significantly reduced (*p <* 0.05) *Ci* and exhibited a trend of first decreasing and then increasing with the increase in SiO_2_-NPs concentration (Fig. 2C). The *Ci* in T3 was decreased (*p* < 0.05) by 7.11% compared to T1, but there was a statistically insignificant difference between T2, T4, and T1.

###  Effect of SiO_2_-NPs on ABA content and LWP of cotton seedlings under the salt and low-temperature dual stress

**Fig. 3:**
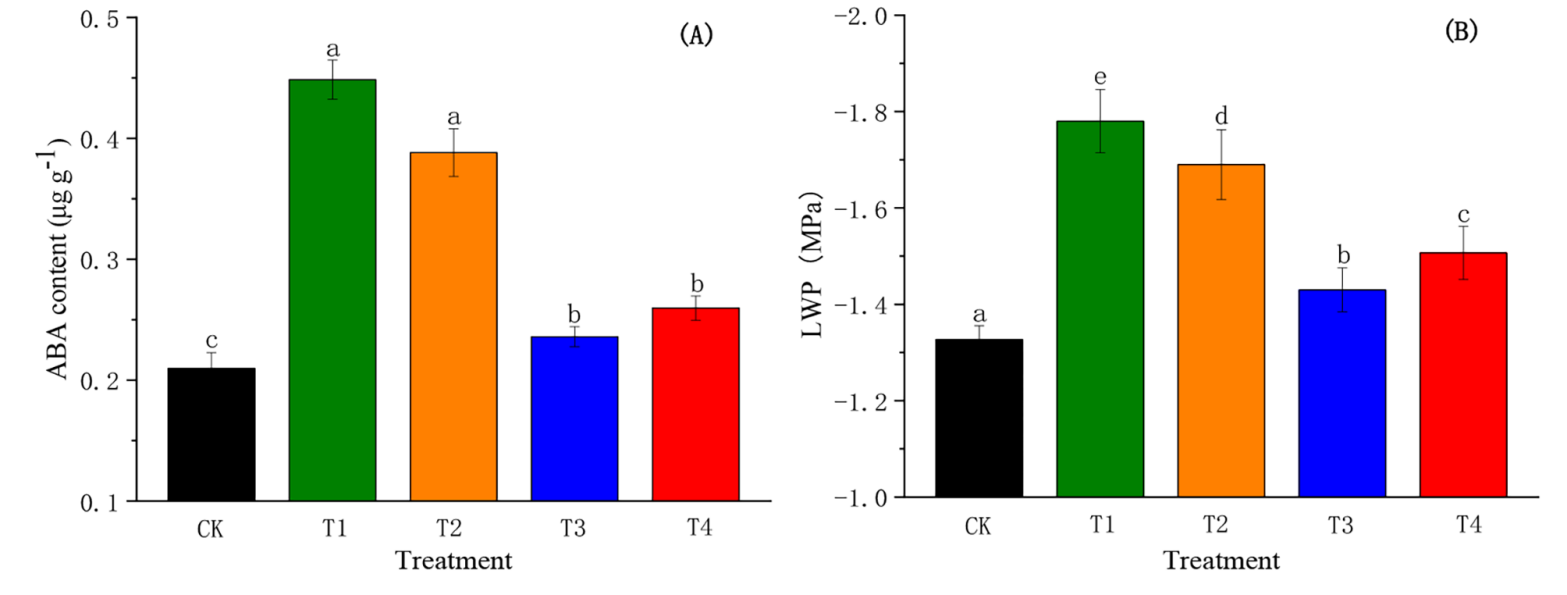
Effects of SiO_2_-NPs on ABA content **(A)** and LWP **(B)** of cotton seedlings under the salt and low temperature dual stress. CK represents the foliar application of 0 mg L^− 1^ SiO_2_-NPs with no stress. T1, T2, T3, and T4 represent the foliar application of 0 mg L^− 1^, 50 mg L^− 1^, 100 mg L^− 1^, and 200 mg L^− 1^ SiO_2_-NPs under the salt and low-temperature dual stress, respectively. Data are mean ± standard deviation (*n* = 3). Different alphabets on top of error bars represent significant differences (*p* < 0.05)

ABA content was significantly reduced by SiO_2_-NPs under the salt and low-temperature dual stress as shown in Fig. 3A. With the increase in SiO_2_-NPs concentration, ABA content showed a trend of first decreasing and then increasing, and T3 obtained the highest reduction of 47.40% compared to T1. Notably, the dual stress caused a substantial decrease in LWP compared to CK as illustrated in Fig. 3B. Conversely, SiO_2_-NPs significantly decreased (*p <* 0.05) the reduction of LWP caused by the dual stress and LWP displayed a trend of first increasing and then decreasing with the increase in SiO_2_-NPs concentration. The LWP in T2, T3, and T4 increased significantly (*p <* 0.05) compared to T1, and the highest increment that appeared in T3 was 19.66%.

### Effects of SiO_2_-NPs on stomatal characteristics of cotton seedlings under the salt and low-temperature dual stress

**Fig. 4:**
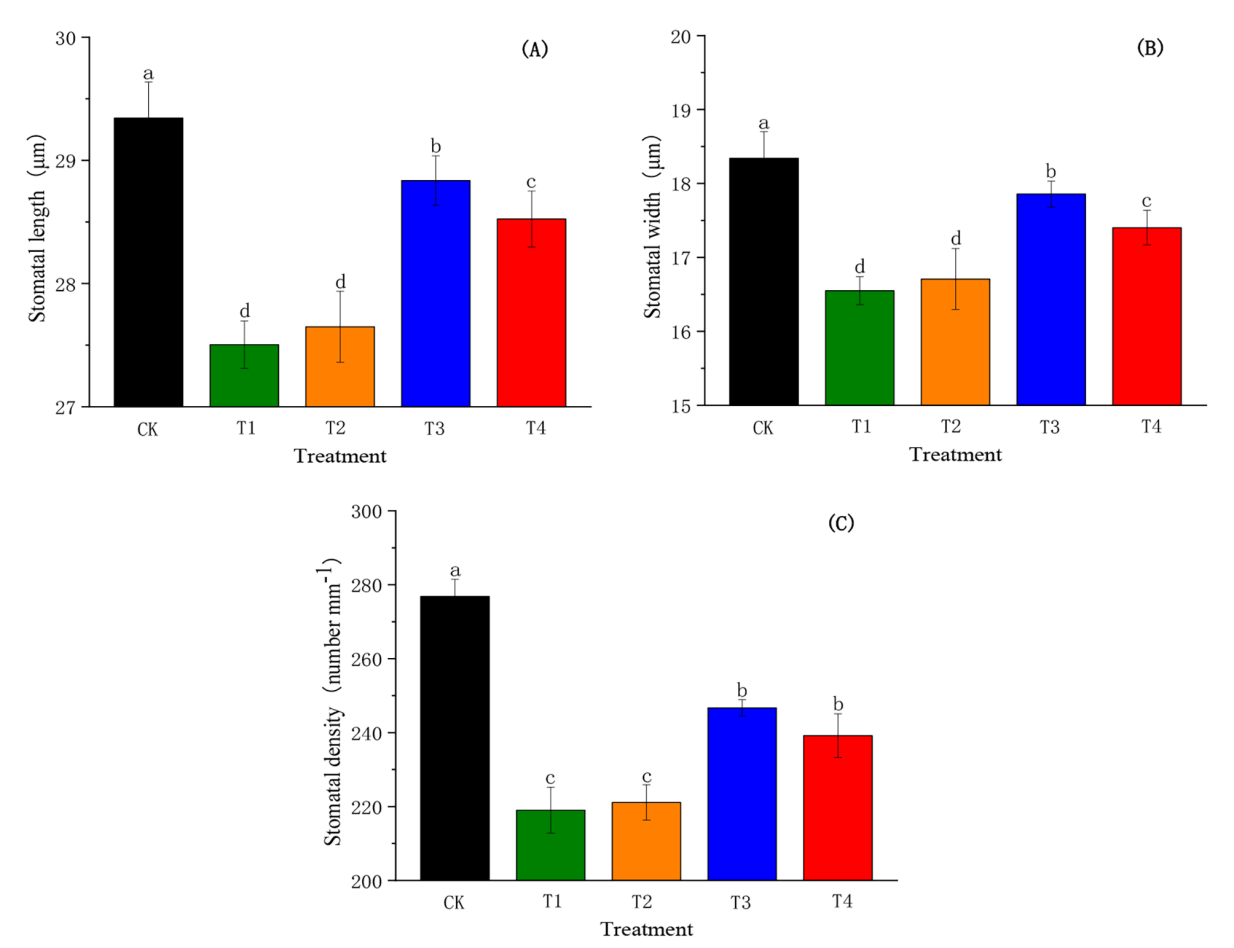
Effects of SiO_2_-NPs on stomatal length **(A)**, stomatal width **(B)**, and stomatal density **(C)** of cotton seedlings under the salt and low temperature dual stress. CK represents the foliar application of 0 mg L^− 1^ SiO_2_-NPs with no stress. T1, T2, T3, and T4 represent the foliar application of 0 mg L^− 1^, 50 mg L^− 1^, 100 mg L^− 1^, and 200 mg L^− 1^ SiO_2_-NPs under the salt and low-temperature dual stress, respectively. Data are mean ± standard deviation (*n* = 3). Different alphabets on top of error bars represent significant differences (*p <* 0.05)

Figure 4 presents the effects of different SiO_2_-NPs concentrations on the stomatal characteristics of cotton seedlings under the salt and low-temperature dual stress. Under the condition of the dual stress without SiO_2_-NPs treatment (T1), the SL, SW, and SD were reduced by 6.27%, 9.77%, and 20.90%, respectively. With the increase in SiO_2_-NPs concentration, the trends of SL, SW, and SD were presented as an increase followed by a decrease. The SL, SW, and SD in T3 and T4 significantly increased (*p <* 0.05) by 1.73%, 2.79%, 2.64%, and 5.13%, 10.90%, 13.62% compared to T1, respectively. However, there was no significant difference between T1 and T2.

### Effects of SiO_2_-NPs on chlorophyll content of cotton seedlings under the salt and low-temperature dual stress

**Fig. 5:**
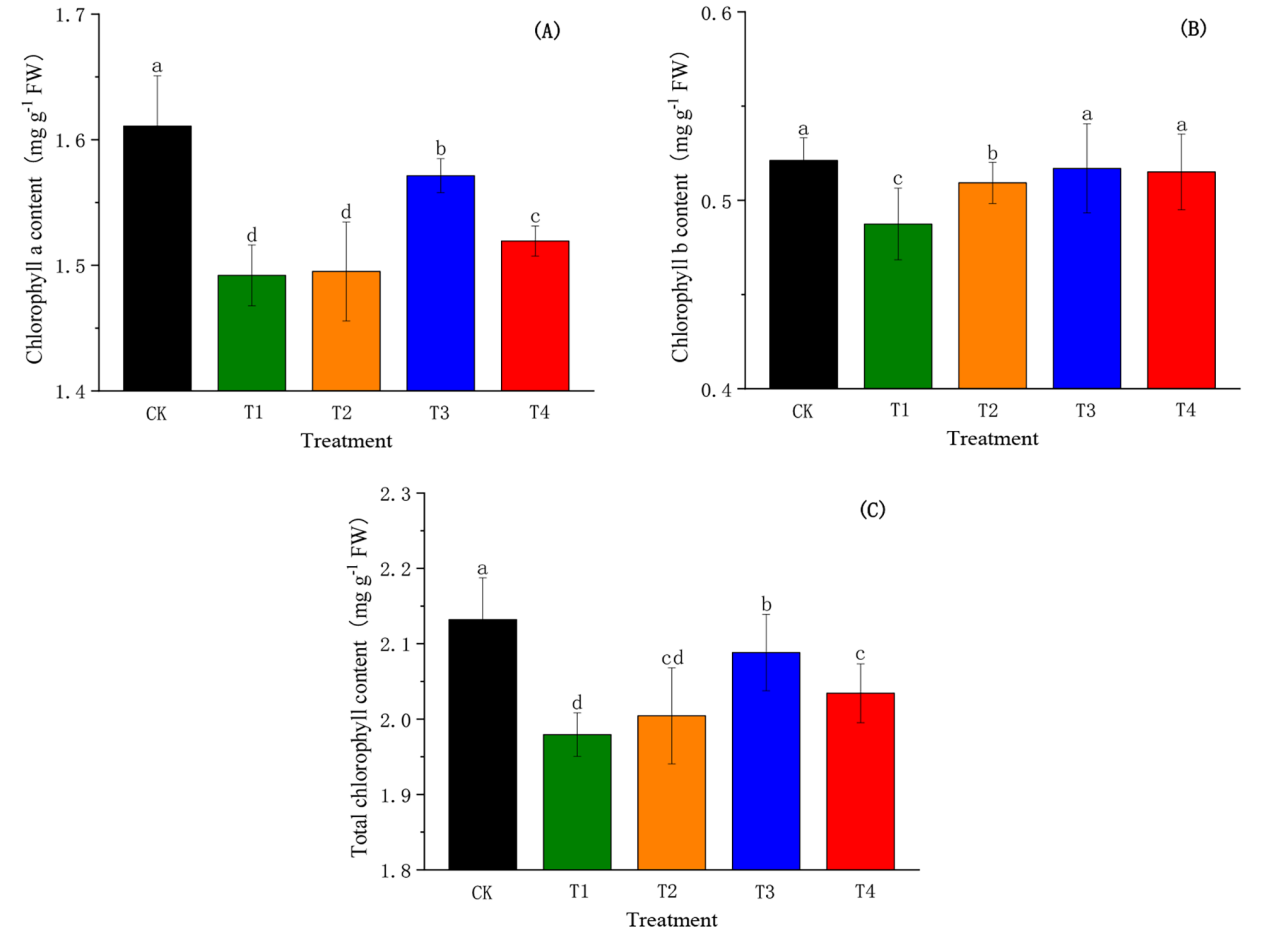
Effects of SiO_2_-NPs on chlorophyll a content **(A)**, chlorophyll b content **(B)**, and total chlorophyll content **(C)** of cotton seedlings under the salt and low temperature dual stress. CK represents the foliar application of 0 mg L^− 1^ SiO_2_-NPs with no stress. T1, T2, T3, and T4 represent the foliar application of 0 mg L^− 1^, 50 mg L^− 1^, 100 mg L^− 1^, and 200 mg L^− 1^ SiO_2_-NPs under the salt and low-temperature dual stress, respectively. Data are mean ± standard deviation (*n* = 3). Different alphabets on top of error bars represent significant differences (*p <* 0.05)

The effects of the various treatments on chlorophyll content are shown in Fig. 5. In this experiment, the salt and low-temperature dual stress showed a decrease of 7.37%, 6.48%, and 7.15% in chlorophyll a, chlorophyll b, and total chlorophyll content, respectively. However, treating plants with SiO_2_-NPs application induced an increase in the content of all pigments. The highest increase was observed in T3 which showed more efficiency than T2 and T4, and chlorophyll a, chlorophyll b, and total chlorophyll content significantly increased (*p* < 0.05) by 5.32%, 6.06%, 5.50% compared to CK, respectively.

### Effects of SiO_2_-NPs on chlorophyll fluorescence parameters of cotton seedlings under the salt and low-temperature dual stress

**Fig. 6:**
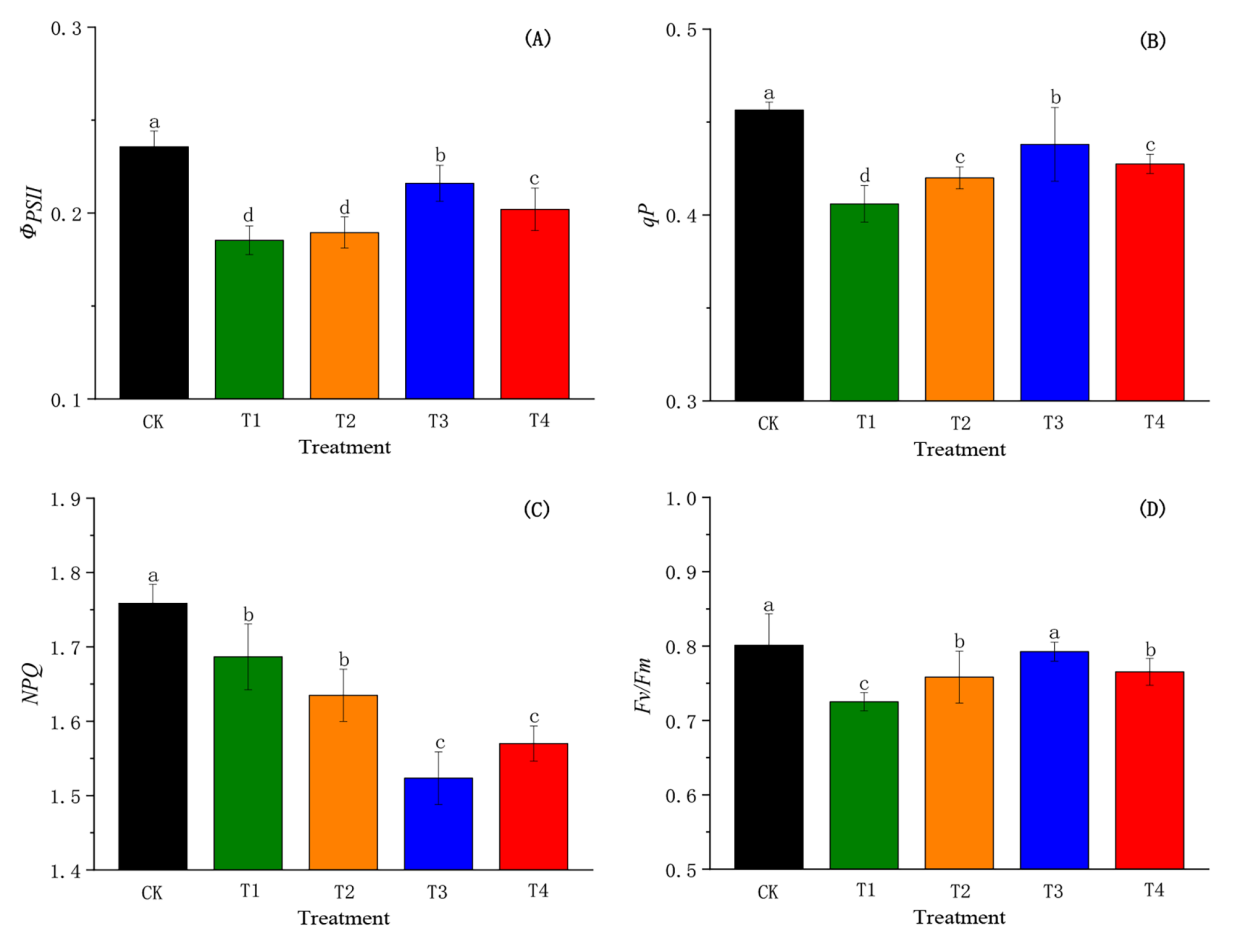
Effects of SiO_2_-NPs on actual photochemical efficiency of photosystem II (*Φ*_*PSI*I_, **A)**, photochemical quenching of variable chlorophyll (*qP*, **B)**, non-photochemical quenching (*NPQ*, **C)**, and maximal photochemical efficiency of photosystem II (*Fv/Fm*, **D)** of cotton seedlings under the salt and low temperature dual stress. CK represents the foliar application of 0 mg L^− 1^ SiO_2_-NPs with no stress. T1, T2, T3, and T4 represent the foliar application of 0 mg L^− 1^, 50 mg L^− 1^, 100 mg L^− 1^, and 200 mg L^− 1^ SiO_2_-NPs under the salt and low-temperature dual stress, respectively. Data are mean ± standard deviation (*n* = 3). Different alphabets on top of error bars represent significant differences (*p <* 0.05)

The responses of *Φ*_*PSII*_, *Fv/Fm*, *NPQ*, and *qP* to SiO_2_-NPs under the salt and low-temperature dual stress are illustrated in Fig. 6. The dual stress generally decreased the *Φ*_*PSII*_, *qP, Fv/Fm*, and *NPQ* of cotton seedlings compared to CK. Contrarily, the *Φ*_*PSII*_, *qP*, and *Fv/Fm* were remarkably increased (*p <* 0.05) by SiO_2_-NPs except for the *NPQ* under the dual stress. Compared to T1, the *Φ*_*PSII*_, *qP*, and *Fv/Fm* in T3 obtained the highest increment by 16.55%, 7.88%, and 9.29%, respectively. The *NPQ* showed a trend of first decreasing and then increasing with the increase in SiO_2_-NPs concentration (Fig. 6C). Compared to T1, the *NPQ* in T3 and T4 significantly decreased (*p <* 0.05) by 9.67% and 6.92%, respectively. However, there was no significant difference between T1 and T2.

### Effects of SiO_2_-NPs on photosynthetic enzymes activities of cotton seedlings under the salt and low-temperature dual stress

**Fig. 7:**
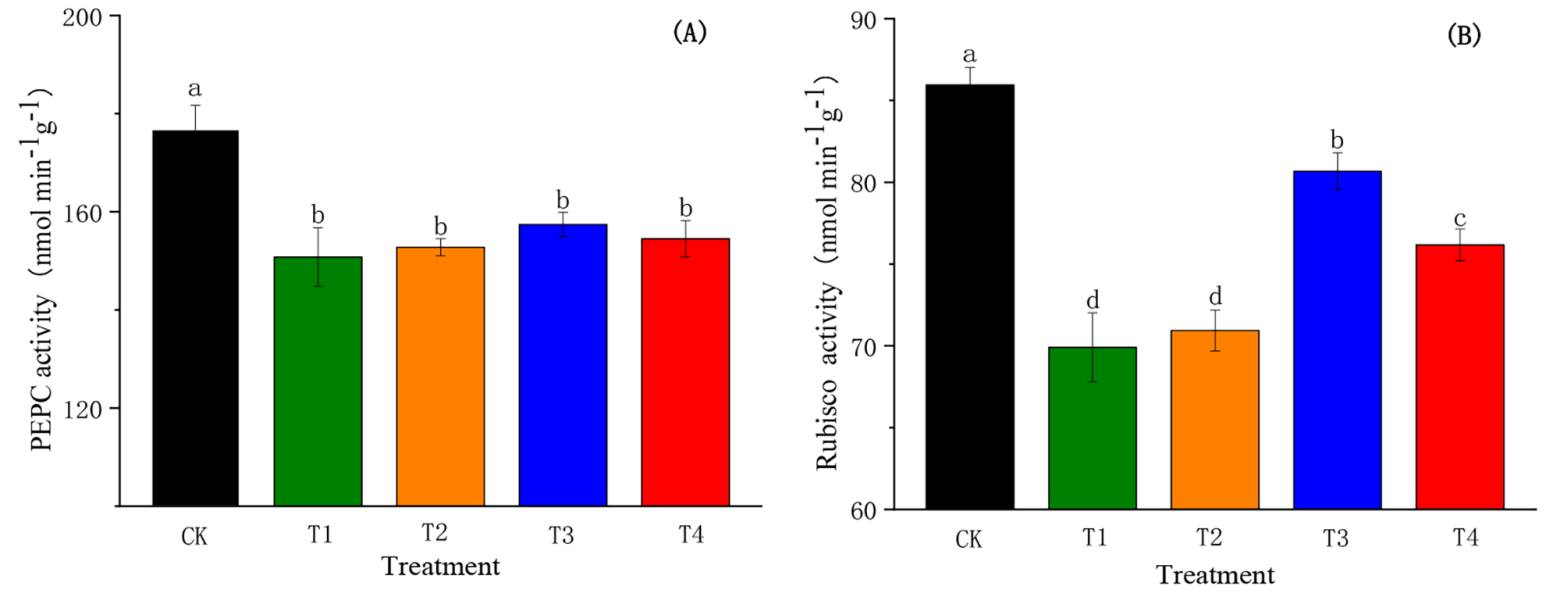
Effects of SiO_2_-NPs on PEPC activity **(A)** and Rubisco activity **(B)** of cotton seedlings under the salt and low temperature dual stress. CK represents the foliar application of 0 mg L^− 1^ SiO_2_-NPs with no stress. T1, T2, T3, and T4 represent the foliar application of 0 mg L^− 1^, 50 mg L^− 1^, 100 mg L^− 1^, and 200 mg L^− 1^ SiO_2_-NPs under the salt and low-temperature dual stress, respectively. Data are mean ± standard deviation (*n *= 3). Different alphabets on top of error bars represent significant differences (*p <* 0.05)

PEPC and Rubisco activities showed significant differences among the different treatments due to the different concentrations of SiO_2_-NPs under the salt and low-temperature dual stress (Fig. 7). The PEPC activity in T1 was significantly reduced (*p <* 0.05) by 14.69% compared to CK, while the SiO_2_-NPs slightly increased the PEPC activity caused by the dual stress (Fig. 7A). Compared to T1, the PEPC activity in T2, T3, and T4 were enhanced by 1.33%, 4.35%, and 2.37%, respectively, but there was no significant difference among T1, T2, T3, and T4. The effect of salt and low-temperature dual stress on Rubisco activity is illustrated in Fig. 7B. The dual stress remarkably reduced (*p* < 0.05) the Rubisco activity by 18.66% compared to CK. Notably, the Rubisco activity in T3 and T4 were significantly improved (*p* < 0.05) by 15.40% and 8.95% compared to T1, respectively. However, there was no significant difference between T2 and T1.

### Effects of SiO_2_-NPs on malondialdehyde content of cotton seedlings under the salt and low-temperature dual stress

**Fig. 8:**
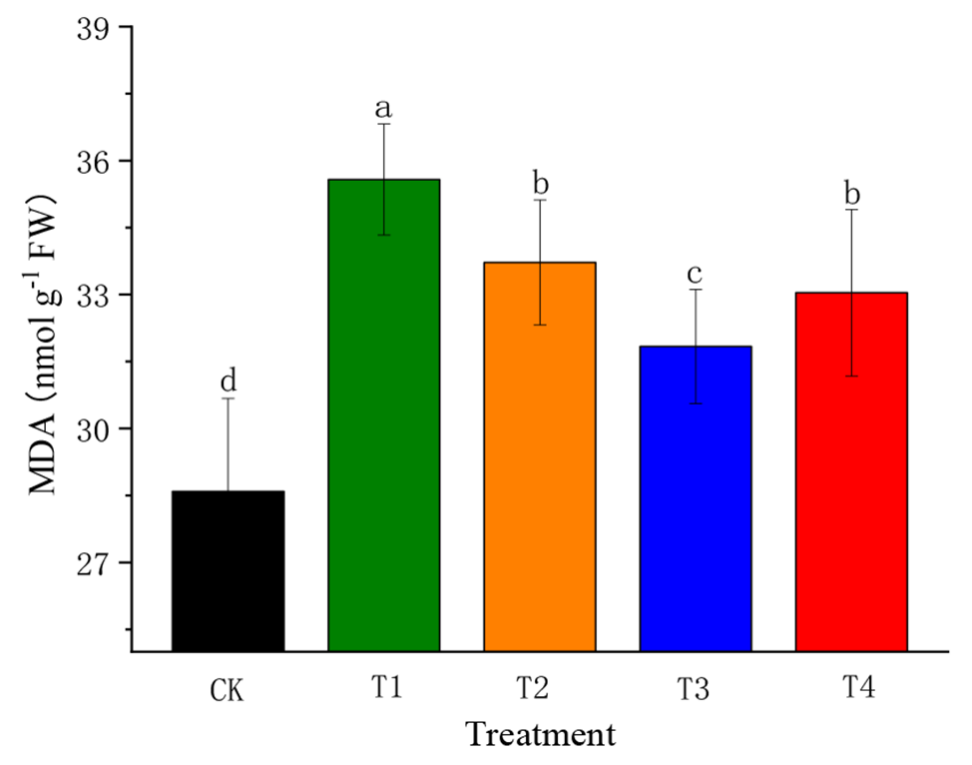
Effect of SiO_2_-NPs on malondialdehyde (MDA) of cotton seedlings under the salt and low temperature dual stress. CK represents the foliar application of 0 mg L^− 1^ SiO_2_-NPs with no stress. T1, T2, T3, and T4 represent the foliar application of 0 mg L^− 1^, 50 mg L^− 1^, 100 mg L^− 1^, and 200 mg L^− 1^ SiO_2_-NPs under the salt and low-temperature dual stress, respectively. Data are mean ± standard deviation (*n* = 3). Different alphabets on top of error bars represent significant differences (*p <* 0.05)

Applying SiO_2_-NPs significantly affected the MDA content in cotton seedlings exposed to the salt and low-temperature dual stress (Fig. 8). The dual stress significantly enhanced (*p* < 0.05) the MDA content by 24.43% compared to CK. The application of SiO_2_-NPs significantly reduced (*p* < 0.05) the MDA content, and decreased monotonously with increasing SiO_2_-NPs concentration. The highest reduction (10.51%) was obtained in T3 compared to T1.

### Correlation analysis and principal component analysis

**Fig. 9:**
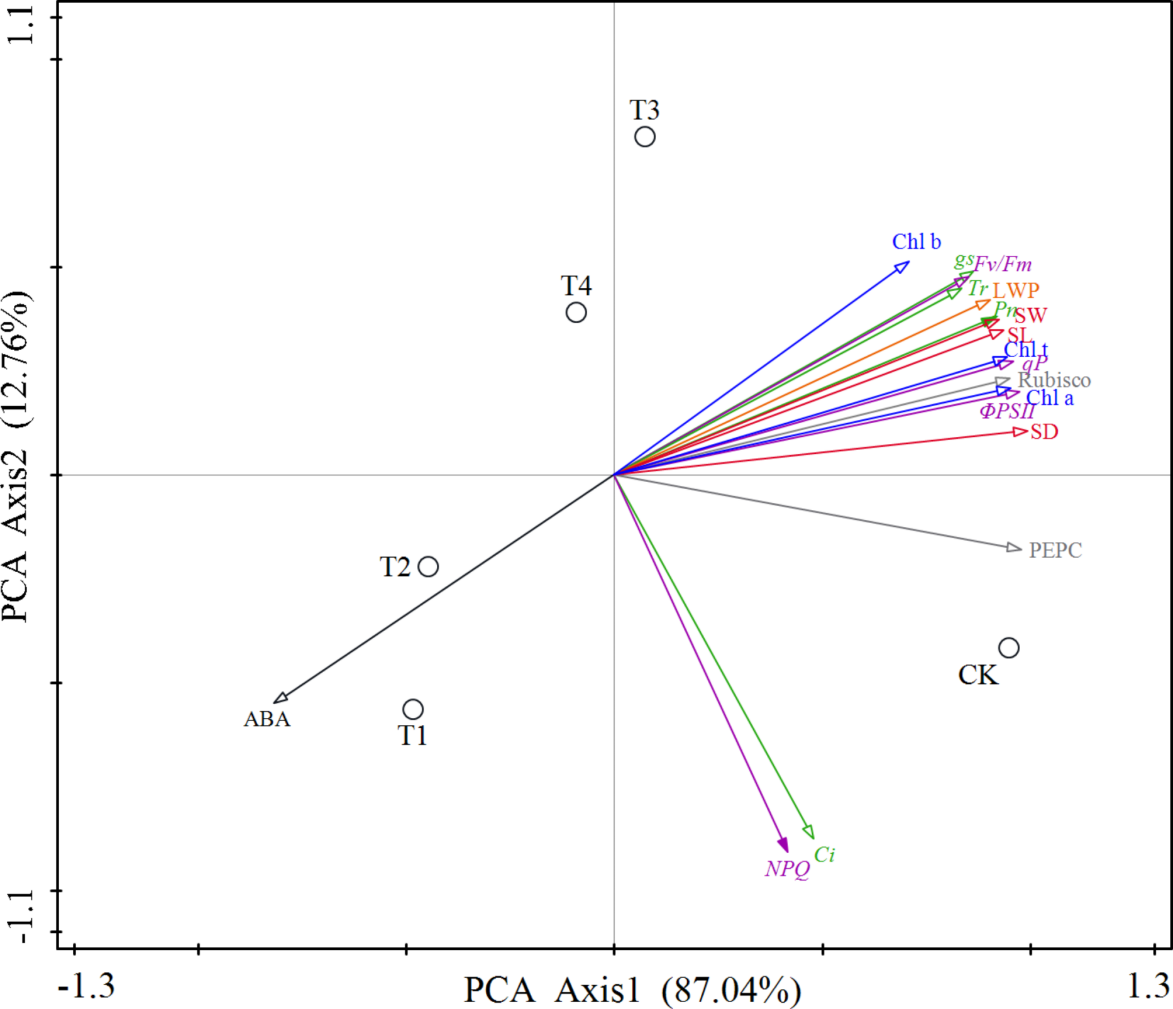
Principal component analyses (PCA) on gas exchange parameters (green lines), stomatal characteristics parameters (red lines), chlorophyll content (blue lines), chlorophyll fluorescence parameters (purple lines), photosynthetic enzyme activities (gray lines), abscisic acid content (ABA, black line), and leaf water potential (LWP, orange line). *Pn* = Photosynthetic rate, *gs* = Stomatal conductance, *Ci* = Intracellular CO_2_ concentration, *Tr* = Transpiration rate, SL = Stomatal length, SW = Stomatal width, SD = Stomatal density, Chl a = Chlorophyll a content, Chl b = Chlorophyll a content, Chl t = Total chlorophyll content, *Φ*_*PSII*_ = Actual photochemical efficiency of photosystem II, *qP* = Photochemical quenching of variable chlorophyll, *NPQ* = Non-photochemical quenching, *Fv/Fm* = Maximal photochemical efficiency of photosystem II. CK represents the foliar application of 0 mg L^− 1^ SiO_2_-NPs with no stress. T1, T2, T3, and T4 represent the foliar application of 0 mg L^− 1^, 50 mg L^− 1^, 100 mg L^− 1^, and 200 mg L^− 1^ SiO_2_-NPs under the salt and low-temperature dual stress, respectively. Values in bracket are percentages explained by the first two components. Data are the means (*n *= 3)

The salt and low-temperature dual stress negatively affected the stomatal exchange parameters, while SiO_2_-NPs alleviated the reduction of *Pn* caused by the dual stress. To better understand the relationships among stomatal exchange parameters, *s*tomatal characteristic parameters, chlorophyll content, chlorophyll fluorescence parameters, photosynthetic enzyme activities, ABA content and LWP, the PCA and Pearson correlation analysis were performed (Fig. 9; Table 1). According to PCA, a relatively higher positive correlation was observed between *Pn* and *gs*, *Tr*, SL, SW, SD, Chl a, Chl b, Chl t, *Φ*_*PSII*_, *qP, Fv/Fm*, Rubisco activity and LWP, and the negative correlation between *Pn* and ABA content at the first component axis, which accounting for 87.04% of the total variation (Fig. 9). It might indicate that SiO_2_-NPs regulated the *Pn* of cotton seedlings by affecting the changes of these parameters under the dual stress. Furthermore, significant correlation coefficients (*p <* 0.05) were obtained between *Pn* and *gs*, *Tr*, SL, SW, SD, Chl a, Chl b, Chl t, *Φ*_*PSII*_, *qP*, *Fv/Fm*, Rubisco activity, LWP, and ABA content, but no significant correlation coefficients were obtained between *Pn* and *Ci*, *NPQ*, and PEPC activity (Table 1).

**Table 1:** The Pearson’s correlation coefficients for gas exchange parameters, stomatal characteristics parameters, chlorophyll content, chlorophyll fluorescence parameters, phosphoenolpyruvate carboxylase (PEPC), ribulose bisphosphate carboxylase/oxygenase (Rubisco), abscisic acid content (ABA), and leaf water potential (LWP).

	*Pn*	*gs*	*Ci*	*Tr*	SL	SW	SD	Chl a	Chl b	Chl t	*Φ*_*PSII*_	*qP*	*NPQ*	*Fv/Fm*	PEPC	Rubisco	ABA	LWP
***Pn***	1	0.965^**^	0.106	0.989^**^	0.987^**^	0.989^**^	0.951^*^	0.948^*^	0.900^*^	0.982^**^	0.971^**^	0.984^**^	0.038	0.970^**^	0.840	0.981^**^	− 0.969^**^	0.996^**^
***gs***		1	− 0.010	0.946^**^	0.988^**^	0.981^**^	0.913^*^	0.923^*^	0.814	0.943^*^	0.936^*^	0.911^*^	− 0.081	0.904^*^	0.746	0.962^**^	− 0.974^**^	0.982^**^
***Ci***			1	− 0.013	0.118	0.146	0.385	0.275	− 0.119	0.204	0.293	0.249	0.997^**^	0.001	0.625	0.221	0.090	0.064
***Tr***				1	0.960^**^	0.960^**^	0.896^*^	0.902^*^	0.949^*^	0.954^*^	0.928^*^	0.963^**^	− 0.079	0.986^**^	0.767	0.942^*^	− 0.977^**^	0.984^**^
**SL**					1	0.996^**^	0.961^**^	0.951^*^	0.838	0.971^**^	0.972^**^	0.958^*^	0.047	0.924^*^	0.833	0.987^**^	− 0.968^**^	0.994^**^
**SW**						1	0.968^**^	0.973^**^	0.828	0.987^**^	0.986^**^	0.970^**^	0.077	0.937^*^	0.854	0.996^**^	− 0.953^**^	0.990^**^
**SD**							1	0.992^**^	0.784	0.993^**^	0.989^**^	0.967^*^	0.319	0.873	0.952^*^	0.982^**^	− 0.873^*^	0.943^*^
**Chl a**								1	0.734	0.989^*^	0.992^**^	0.959^*^	0.214	0.908^*^	0.904^*^	0.988^**^	− 0.861	0.936^*^
**Chl b**									1	0.825	0.784	0.877	− 0.179	0.936^*^	0.634	0.798	− 0.915^*^	0.889^*^
**Chl c**										1	0.993^*^	0.986^**^	0.140	0.956^*^	0.888^*^	0.993^**^	− 0.913^*^	0.969^*^
***Φ***_***PSII***_											1	0.981^**^	0.228	0.921^*^	0.924^*^	0.996^**^	− 0.894^**^	0.961^**^
***qP***												1	0.185	0.962^**^	0.909^*^	0.975^**^	− 0.913^**^	0.966^**^
***NPQ***													1	− 0.059	0.572	0.154	0.162	− 0.006
***Fv/Fm***														1	0.769	0.927^*^	− 0.931^**^	0.953^**^
**PEPC**															1	0.891^*^	− 0.700	0.811
**Rubisco**																1	− 0.923^*^	0.976^**^
**ABA**																	1	− 0.983^**^
**LWP**																		1

Note: *Pn* = Photosynthetic rate, *gs* = Stomatal conductance, *Ci* = Intracellular CO_2_ concentration, *Tr* = Transpiration rate, SL = Stomatal length, SW = Stomatal width, SD = Stomatal density, Chl a = Chlorophyll a content, Chl b = Chlorophyll a content, Chl t = Total chlorophyll content, *Φ*_*PSII*_ = Actual photochemical efficiency of photosystem II, *qP* = Photochemical quenching of variable chlorophyll, *NPQ* = Non-photochemical quenching, *Fv/Fm* = Maximal photochemical efficiency of photosystem II. ^*^ and ^**^ indicate significance levels of *p <* 0.05 and *p <* 0.01, respectively

## Discussion

Photosynthesis is the basis of plant growth and strongly links to leaf water content because the normal water content in the leaves assists the stomata open [44]. In our study, the salt and low-temperature dual stress caused significant and severe reductions in plant height, leaf area, aboveground biomass, and a decrease in *Pn*, *gs*, and LWP. This might be because osmotic stress caused by salt and low-temperature dual stress greatly decreased the absorption of water by the root system [45], which reduced inter- and intracellular water levels and stomatal aperture [46]. Another similar research reported that salt and low-temperature stress generated some chemical signals which induced stomatal closure and further reduced water loss [47], therefore inhabited the photosynthetic efficiency and plants growth. In the current study, SiO_2_-NPs application significantly increased the plant height, leaf area, aboveground biomass, *Pn*, *gs*, and LWP of cotton seedlings, which indicated that SiO_2_-NPs decreased the damage of osmotic stress. On the one hand, SiO_2_-NPs might induce the increase in osmotic regulatory substances to increase osmotic potential introduced by the salt and low-temperature stress [48]. In addition, SiO_2_-NPs could regulate aquaporin (AQP) abundance to facilitate water uptake and transport across cell membranes [49]. Our results showed that the growth of cotton seedlings increased with increasing SiO_2_-NPs concentration up to 100 mg L^− 1^ (T3) and then decreased with further increases in SiO_2_-NPs concentration, which might indicate that high SiO_2_-NPs concentrations had cytotoxicity and inhibitory effects on plant growth [50].

Stomata serve as an important channel for water and gas exchange between plants and the external environment [51]. Existing literature has documented that stomatal conductance was associated with stomata size, pore area and stomatal density [52], which was consistent with the results of our study (Table 1). A previous study suggested that the stomata aperture was a direct response to the leaf water status [53]. Liu et al. also found Si could increase *Pn* due to the enhancement in *gs* resulting from an improvement of the leaf water content [49]. Additionally, researchers had reported that Si-mediated the decrease in ABA content was one of the key approaches affecting photosynthesis efficiency. It might be because Si restricted the gene expression mediated in the ABA synthesis pathway, which maintained the stomatal aperture to increased osmotic stress tolerance [54]. Furthermore, some studies have reported that *gs* was co-regulated by ABA content and LWP under adversity stresses [55, 56]. Similar results were observed in the current study that there was a high correlation between ABA content, LWP, and *gs* (Table 1).

Salt and low-temperature stress cause the overproduction of ROS inside the plant cell, which alter the metabolic and oxidative homeostasis of plant cells, hence promoting membrane lipid peroxidationlead (LPO). As MDA is a major product of LPO in plants, it is a key indicator of the oxidative stress [57, 58]. In this experiment, the MDA content increased under the dual stress (Fig. 8), which was consistent with the findings of Liu et al. on the MDA contents of Bermuda grass [59]. While, SiO_2_-NPs alleviated the deleterious consequences, this mitigation was not only attributed to the increase in antioxidant enzyme activities, but also resulted from non-enzymatic mechanisms such as increased proline and glutathione contents [60, 61].

Due to the defect of biochemical properties in plants grown under stressful conditions, the photosynthesis process and its efficiency will be affected and inhibited [33, 38]. Our results support this statement as chlorophyll a, chlorophyll b and total chlorophyll content significantly diminished in response to the salt and low-temperature dual stress (Fig. 5). The reduction in chlorophyll content could be a result of the damage to chloroplast structure and a restriction in their biosynthesis [62]. However, SiO_2_-NPs ameliorate the reduction in chlorophyll content, similar results were found by Haghighi and Pessarakli who also reported enhanced chlorophyll synthesis and photosynthesis in SiO_2_-NPs treated Solanum lycopersicum seedlings [63]. In addition, one mechanism about Si protecting photosynthetic pigments under stressful conditions was reported that Si could form a binary film at the cell wall to maintain the structural stability of cells [64].

Chlorophyll fluorescence parameters reflect the characteristics of plant absorption, transfer, dissipation and light energy distribution, which are marked as effective probes for investigating photosynthesis in plants under adversity stresses [65, 66]. Changes in chlorophyll fluorescence parameters accurately characterize the photosynthetic potential of plants and the degree of photosystem damage [67]. *Fv/Fm* and *Φ*_*PSII*_ represent the efficiency of PSII in converting absorbed light energy into chemical energy [21], *qP* reflects the photosynthetic activity of PSII reaction center [68]. Our results demonstrated that *Fv/Fm, Φ*_*PSII*_, and *qP* were significantly lower in T1 compared to CK, which might be implied that a marked photo-inhibition was occurred here [39]. While, SiO_2_-NPs increased *Φ*_*PSII*_, *Fv/Fm*, and *qP* (Fig. 6) under the dual stress. One reason might be that SiO_2_-NPs enhanced light absorbance, which could inhabit the chloroplast ageing and improve the photosynthetic period of chloroplast [69]. Moreover, SiO_2_-NPs might enhance oxygen transport and the enzyme activity of carbohydrate metabolism to improve photosynthesis efficiency [70]. *NPQ* reflects the ability of plants to dissipate excess light energy in the form of heat which cannot be used for the transfer of photosynthetic electrons and the damage degree of photosynthetic apparatus [71]. Our results proved that SiO_2_-NPs reduced *NPQ* under the salt and low-temperature dual stress, which could be explained by the fact that SiO_2_-NPs decreased the dissipation of excess light energy and alleviated the damage of photosynthetic apparatus [72].

Rubisco and PEPC are key enzymes affecting photosynthesis in plants [73]. Rubisco catalyzes the carboxylation and oxygenation reactions of ribulose-1,5-bisphosphate (RuBP) and controls photosynthetic carbon metabolism and photorespiration in plants [74]. We found Rubisco activity significantly decreased when suffering from the salt and low-temperature dual stress, it might be because of the increase in chloroplast protrusion to release vesicles containing Rubisco (Rubisco-containing body) which one of the pathways of Rubisco exclusion from chloroplasts [75]. However, the reduction of Rubisco activity declined after spraying SiO_2_-NPs (Fig. 7), which was consistent with the findings of Pereira et al. who found Si application increased the activity of Rubisco that was involved in the Si-induced regulation of photosynthesis [76]. PEPC is closely related to the ability of leaves to fix CO_2_ and remobilize CO_2_ released by respiration in C3 plants [77]. In this study, PEPC significantly decreased under the salt and low-temperature dual stress, but there was no significant difference between different treatments after spraying SiO_2_-NPs (Fig. 7). Meanwhile, our findings proved that *Pn* was significantly correlated with Rubisco activity but not with PEPC activity (Table 1), which also indicated that SiO_2_-NPs might mainly by regulated a higher Rubisco activity under the salt and low-temperature dual stress to promote photosynthesis.

Stomatal and non-stomatal factors influencing photosynthesis are generally decided by the relationship between *gs* and *Ci* [78]. Our results showed that *gs* and *Ci* in T1 were significantly lower than CK, which suggested that the salt and low-temperature dual stress affected the diffusion process of CO_2_ by inducing stomatal closure, which resulted in a significant decrease in the concentration of CO_2_ diffusing from the external environment to the leaf through the stomata [24]. However, there was a significant increase in *gs* but not in *Ci* after spraying SiO_2_-NPs, which implied that the reduction of *Pn* was limited by stomatal and non-stomatal factors. In the current study, the positive correlations were observed between *Pn* and *gs*, SL, SW, SD, chlorophyll content, chlorophyll fluorescence parameters (*Fv/Fm*, *Φ*_*PSII*_, *qP*), and Rubisco activity (Table 1), and T3 had the most positive influences on these parameters. This result might reveal that SiO_2_-NPs at the concentration of 100 mg L^− 1^ could optimally regulate stomatal opening and closing, enhance the photochemical efficiency and photosynthetic activity of PSII reaction centers and increase Rubisco activity to obtain the highest *Pn*.

## Conclusion

The salt and low-temperature dual stress significantly reduced LWP, *gs*, chlorophyll content, PSII activity, photosynthetic enzyme activity, and enhanced MDA content, which caused the decrease in cotton seedling photosynthesis and growth. While SiO_2_-NPs application alleviated the deleterious consequences of the dual stress. The positive correlations were observed between *Pn* and *gs*, SL, SW, SD, chlorophyll content, *Fv/Fm*, *Φ*_*PSII*_, *qP*, and Rubisco activity. It indicated that SiO_2_-NPs enhanced the photosynthesis of cotton seedlings by regulating the stomatal state, improving the light energy utilization efficiency and electron transport activity of PSII, and inducing the increase of Rubisco activity to enhance the carbon assimilation capacity under the salt and low-temperature dual stress. According to our results, SiO_2_-NPs at the concentration of 100 mg L^− 1^ could be recommended to mitigate the damage in cotton seedling growth under the salt and low-temperature dual stress. However, there was only one combined treatment, and the effects of SiO_2_-NPs on the ion transport, osmotic regulation, antioxidant defense, signal conduction, and gene expression are still unclear. Further studies are required under a wide range of salt and low-temperature combined treatments to examine the biochemical and molecular mechanism of SiO_2_-NPs, to provide deeper insights into the effects of SiO_2_-NPs on plant growth under multiple stress.

## Data Availability

The data that support the findings of this study are available from the corresponding author upon reasonable request.

## Abbreviations

*Fv/Fm*: Maximum photochemical efficiency
*Φ*_*PSII*_: Actual photochemical efficiency
*qP*: Photochemical quenching of variable chlorophyll
*NPQ*: Non-photochemical quenching
Rubisco: Ribulose-1,5-bisphosphate carboxylase/oxygenase
PEPC: Phosphoenolpyruvate carboxylase
SiO_2_-NPs: Silicon nanoparticles
ABA: Abscisic acid
LWP: Leaf water potential
*Pn*: Photosynthetic rate
*Ci*: Intracellular CO_2_ concentration
*gs*: Stomatal conductance
*Tr*: Transpiration rate
SD: Stomatal dendity
SW: Stomatal width
SL: Stomatal length
MDA: Malondialdehyde
Chl a: Chlorophyll a content
Chl b: Chlorophyll b content
Chl t: Total chlorophyll content
